# Case report: Multicentric Castleman disease as a manifestation of immune reconstitution inflammatory syndrome in Malawi

**DOI:** 10.3389/fonc.2022.969135

**Published:** 2022-12-12

**Authors:** Matthew S. Painschab, Marriam Mponda, Tamiwe Tomoka, Coxcilly Kampani, Fred Chimzimu, Yuri Fedoriw, Satish Gopal

**Affiliations:** ^1^ University of North Carolina (UNC) Project Malawi, Lilongwe, Malawi; ^2^ Lineberger Comprehensive Cancer Center, University of North Carolina, Chapel Hill, NC, United States; ^3^ Center for Global Health, National Cancer Institute, Rockville, MD, United States

**Keywords:** multicentric castleman disease, HIV, IRIS, KSHV, malawi, africa

## Abstract

**Introduction:**

Multicentric Castleman disease (MCD) is a lymphoproliferative disorder characterized by systemic inflammation, lymphadenopathy, and cytopenias. MCD caused by Kaposi sarcoma herpesvirus (MCD-KSHV) frequently arises in the context of HIV. It can be associated with immune reconstitution inflammatory syndrome (IRIS), but MCD-IRIS is rarely reported in sub-Saharan Africa (SSA) where HIV and KSHV infection are common.

**Case description:**

A 36-year-old woman in Malawi with HIV on antiretroviral therapy (ART) for nine years presented with fatigue, weight loss, and lymphadenopathy. Lymph node biopsy was consistent with HIV lymphadenitis without evident KSHV-MCD and HIV RNA was 4,244 copies/mL. She switched to second-line ART and returned four months later with worsening lymphadenopathy, fever, night sweats, weight loss, and anemia. A repeat lymph node biopsy demonstrated unequivocal KSHV-MCD features not present on the original biopsy. Her repeat HIV viral load was undetectable and she received chemotherapy with subsequent remission on continued ART for 24 months.

**Discussion:**

This is among the first reported cases of MCD-IRIS from SSA, which has implications for a region where HIV and KSHV are highly prevalent. MCD-IRIS may contribute to early mortality after ART initiation in SSA, and increased awareness alongside improved diagnostic and treatment capacity are needed.

## Introduction

Multicentric Castleman disease (MCD) is a lymphoproliferative disorder characterized by systemic inflammation, lymphadenopathy, anemia and thrombocytopenia (1, 2). MCD is defined by histopathologic findings but contains a spectrum of disorders, including the subtype caused by Kaposi sarcoma herpesvirus (KSHV) which primarily occurs in HIV-infected individuals (3). In immunocompetent hosts, primary KSHV infection is followed by rapid viral suppression similar to other herpesviruses. However, in immunosuppressed individuals, chronic viral reactivation can lead to Kaposi sarcoma (KS), KSHV-MCD, or primary effusion lymphoma (3, 4). KSHV seroprevalence varies widely but is highest in sub-Saharan Africa (SSA) with prevalence 40% in many countries, compared to 10% in the United States and Europe (4, 5). Our group in Malawi has previously described clinical features and outcomes for a large prospective cohort of MCD patients (6–8). Based on our experience, MCD appears to be quite common in Malawi, comprising 15% of lymphoproliferative disorders among persons living with HIV (PLWH) and 7% of all lymphoproliferative disorders, suggesting MCD may be substantially underdiagnosed in other SSA settings.

Immune reconstitution inflammatory syndrome (IRIS) is a syndrome characterized by paradoxical worsening or unmasking of any neoplastic, infectious, or inflammatory disease after initiation of antiretroviral therapy (ART). Though IRIS definitions vary, all contain some combination of a decrease in HIV viral load and worsening disease in the first few months after starting ART (9–12). A significant proportion of early deaths in patients starting ART for HIV are attributed to IRIS, especially in patients with low CD4 count (9). Although IRIS has been well described, including in SSA, reports of MCD-IRIS are rare. Here we describe in detail a patient with MCD-IRIS diagnosed and treated in Malawi.

## Case description

**Figure 1:**
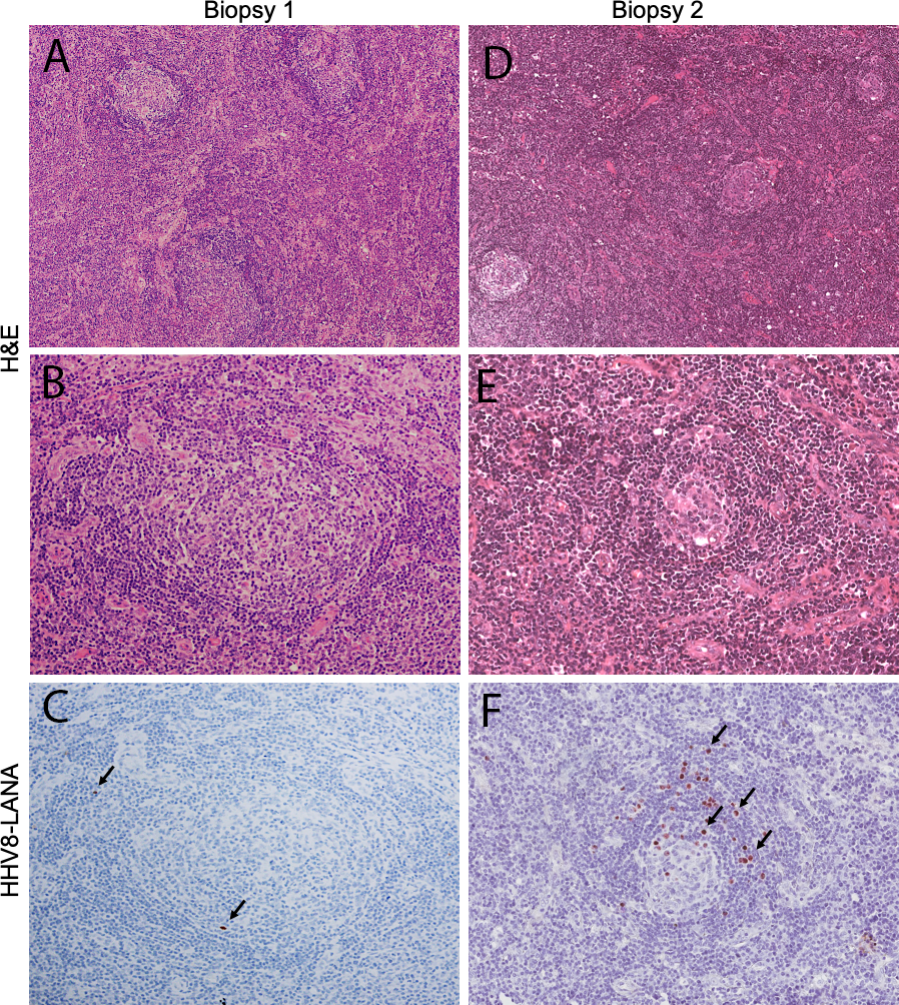
Histologic features of tissue biopsies. The first biopsy (left column) shows morphologic features that are expected on the spectrum of HIV lymphadenopathy, with generally uniform appearing germinal centers and paracortical hyperplasia (**A**; objective magnification x4), The cellular architecture of the germinal centers is preserved (**B**; objective magnification x10) and only rare, small lymphocytes are HHV8-LANA-positive (**C**; arrows; objective magnification x10). In contrast the second biopsy (right column) demonstrates small germinal centers (**D**; objective magnification x4), showing stromal hyalinizaiton and decreased cellularity (**E**; objective magnification x10). Numerous HHV8-LANA-positive plasmablasts rim the atretic follicles (**F**; arrows; objective magnification x10), diagnostic of MCD.

A 36-year-old Malawian woman with HIV diagnosed nine years prior, on ART (tenofovir, lamivudine, and efaverinz), presented with a one-year history of malaise, fatigue, subjective fever, and weight loss. She had no other relevant family, medical, or social history. Over the previous eight months, she had developed progressive cervical and axillary lymphadenopathy. The largest lymph node was two cm in diameter. There was no evidence of KS on examination. Laboratory examination was significant for hemoglobin 11.4 g/dL, platelets 157 x 10^3^/uL, HIV RNA 4,244 copies/mL and CD4 count 62 cells/mm^3^ (**Table 1**). A chest x-ray and abdominal ultrasound were normal. An excisional lymph node biopsy (**Figure 1**) showed non-specific histologic features compatible with HIV-lymphadenopathy, with generally uniform geminal centers and prominent paracortical hyperplasia. Scattered, small lymphoid cells showed expression of latency-associated nuclear antigen-1 (LANA-1), but neither histology nor staining pattern were typical of KSHV-MCD and plasma KSHV DNA was detectable but 500 copies/mL. Similarly, there was no evidence of lymphoma or KS. Diagnostic challenges included lack of access to advanced imaging such as CT scan and PET scan as well as access to only a limited immunohistochemical antibody profile. Given documented ART virologic failure, the patient was switched to second-line ART (zidovudine, lamivudine, atazanavir/ritonavir).

**Table 1:** Timeline of laboratory and clinical characteristics as described in the case.

Characteristic	Initial Visit	Before Chemotherapy	After Chemotherapy
Weight (kg)	60.4	56.5	63.0
Hemoglobin (g/dL)	11.4	8.7	12.0
Platelets (x 10^3^/uL)	157	189	233
HIV Viral Load (copies/mL)	4,244	Undetectable	Undetectable
CD4 Count (cells/mm^3^)	62	Not assessed	193
KSHV Viral Load (copies/mL)	Detectable, 500	Not assessed	Undetectable

Four months later, she returned with worsening diffuse lymphadenopathy, fatigue and myalgias. Her body weight had decreased from 60.4 kg to 56.5 kg. Her hemoglobin had decreased to 8.7 g/dL and her platelet count was 189 x 10^3^/uL (**Table 1**). Her HIV viral load was now undetectable. Repeat chest x-ray and abdominal ultrasound were normal. A differential diagnosis of IRIS secondary to tuberculosis, disseminated fungal infection, or lymphoma was considered. We also considered Kaposi sarcoma inflammatory cytokine syndrome (KICS), an inflammatory syndrome resembling MCD driven by KSHV, but KICS would require an absence of other pathology on lymph node biopsy. A repeat excisional biopsy (**Figure 1**) showed features strongly consistent with KSHV-MCD including variably involuted germinal centers rimmed by LANA-1 positive plasmablasts and did not demonstrate tuberculosis, fungal infection, or other lymphoma.

The patient was diagnosed with MCD-IRIS and treated with eight cycles of cyclophosphamide 750 mg/m^2^, vincristine 1.4 mg/m^2^, and prednisone 60 mg/m^2^, every three weeks. She had no serious adverse events during treatment and tolerated treatment well. After eight cycles, lymphadenopathy and systemic symptoms completely resolved, weight increased to 63.0 kg, hemoglobin increased to 12.0 g/dL, platelets increased to 233 x 10^3^/uL, HIV viral load remained undetectable, and CD4 count increased to 191 cells/uL (**Table 1**). KSHV viral load was undectable after chemotherapy. The patient has subsequently remained in remission for 24 months.

## Methods

After informed consent, the patient was enrolled in the Kamuzu Central Hospital Lymphoma Study (NCT02835911) prospective cohort described previously (6). All diagnoses were pathologically confirmed by histology and immunohistochemistry supported by real-time telepathology between pathologists in Malawi and the United States.

## Discussion

We describe MCD-IRIS occurring after second-line ART in Malawi, a phenomenon which has been infrequently reported in SSA. Strengths of this case study are the prospective, comprehensive patient evaluation and follow-up available. Weaknesses include lack of access to diagnosic testing including real time KSHV viral load testing. Aspects of this case have broader implications for the control of KSHV-associated diseases in SSA.

First, KSHV-MCD may become more and more common across SSA as ART access improves. KS tends to develop in patients with severe immunosuppression characterized by low CD4 T-cell counts and poorly controlled HIV, or T-cell dysfunction from immunosuppressive medications in solid organ transplant recipients. However, KSHV-MCD tends to present in patients with HIV on ART for many years, with well-preserved CD4 counts, and some studies have suggested increasing MCD incidence among HIV-infected individuals with increasing access and earlier application of ART (4, 13, 14).

Next, to our knowledge, this is among the first documented cases of MCD-IRIS from SSA (15–18) and adds to an emerging literature related to KSHV-MCD in the region (6, 19). Given the frequency of HIV and KSHV infection throughout SSA, our report raises the possibility that MCD-IRIS may contribute to continued excess early mortality after ART initiation, including for patients without clinically evident KSHV-associated disease manifestations before ART initiation. For example, a study in Botswana found 28% mortality and 9% lost to follow-up in the first year after ART initiation (20). These deaths likely have multifactorial causes but 12% were attributed to anemia/pancytopenia and an additional 29% of deaths were from “unknown cause.” Our report suggests that some early deaths may be attributable to unrecognized MCD-IRIS or KICS, a closely related disease characterized by cytokine storm in the presence of KS but without pathologic findings of MCD (21). Supporting this hypothesis, a recent study found a significantly higher risk of mortality from KS-IRIS among KS patients in South Africa compared to the United Kingdom with risk being especially high among those with an elevated blood KSHV load (11, 22). KSHV-MCD and KICS are likely not diagnosed in many SSA settings given protean systemic manifestations with more limited access to lymph node biopsy and LANA immunohistochemistry. More information is needed on the early causes of death after ART in SSA in order to develop comprehensive evidence-based strategies to address the early death rate.

Finally, MCD may emerge as an important complication in the use of checkpoint inhibitor immunotherapy for cancer in PLWH. Recent development and clinical testing of immune checkpoint inhibitors have led to numerous indications in high-income countries (23). Access to such agents in SSA remains extremely limited, and registrational trials in high-income countries have typically excluded patients with HIV and/or been conducted in settings where KSHV prevalence is low (24). However, despite the small number of PLWH who have been treated on clinical trials, MCD has been reported after checkpoint inhibitors in patients with HIV-associated KS, including a patient who died of this complication (25). In SSA where KSHV infection is often co-occurring with HIV, MCD may therefore be observed as an important treatment-related toxicity for cancer patients receiving immune checkpoint inhibitor therapy as access to these agents increases. This highlights the continued need for rigorous clinical evaluation of anticancer medicines when applied in previously unstudied populations and contexts, even when these agents have received regulatory approvals in the United States or Europe.

In summary, with increasing access to ART across SSA, increased awareness of MCD is needed, including as a possible IRIS manifestation after ART initiation. Expanded diagnostic and treatment capacity are also needed to mitigate this potentially unrecognized and emerging contributor to morbidity and mortality in several distinct patient populations in SSA.

## Patient perspective

“When I was diagnosed with cancer almost at the same time I was diagnosed with HIV, I just accepted the situation and I did not have any worries. Doctors explained to me about my situation and I was ready to start treatment. During my course of treatment with ARVs and cancer medications, I was experiencing different symptoms like weight loss, dizziness and loss of appetite. Along the way my ARVs got switched to a new regimen because I was having some blood derangements. Other than these, there were no general things affecting my life.

After finishing cancer treatment, everything got improved. I no longer experience the above symptoms. I am able to do house chores except that sometimes I feel short of breath after walking a long distance. In general, my life and health got improved greatly after receiving chemotherapy. I would like to encourage others that if they get diagnosed with this type of cancer they should not be worried because it can get cured.

Lastly, I would like to thank you doctors to continue helping other patients the same way you helped me so that they can also be cured and be happy as I am.”

## Data availability statement

The original contributions presented in the study are included in the article/supplementary material. Further inquiries can be directed to the corresponding author.

## Ethics statement

The studies involving human participants were reviewed and approved by Malawi National Health Sciences Research Committee University of North Carolina Institutional Review Board. The patients/participants provided their written informed consent to participate in this study. Written informed consent was obtained from the individual(s) for the publication of any potentially identifiable images or data included in this article.

## Author contributions

MP conceived and designed the study, collected data, and wrote the paper. MM, YF, CK, FC, and TT collected data. SG conceived and designed the study. All authors approved the final version of the manuscript.
